# Editorial: Nutrition as a pharmacological approach to metabolic disorders and ageing

**DOI:** 10.3389/fphar.2025.1770988

**Published:** 2026-01-13

**Authors:** Chiara Ruocco, Amit Kumar Singh, Enzo Nisoli, Maurizio Ragni

**Affiliations:** 1 Center of Study and Research on Obesity, Department of Medical Technologies and Translational Medicine, University of Milan, Milan, Italy; 2 Department of Botany, BMK Govt. Girls College Balod, Chattisgarh, India

**Keywords:** ageing, cancer, essential amino acids, nutrients, obesity

## Introduction

**FIGURE 1:**
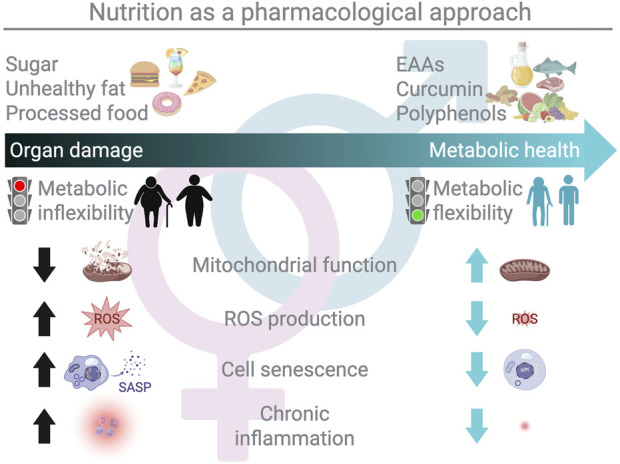
Nutrition as a pharmacological approach: from organ damage to health by restoring metabolic flexibility. The figure illustrates nutrients as metabolic modulators that directly influence metabolic flexibility (i.e., the ability to switch efficiently between fuel sources in response to changing metabolic demands) with sex-specific differences. Diets high in sugars, unhealthy fats, and ultra-processed foods (e.g., a Western-style diet) promote mitochondrial dysfunction, increased reactive oxygen species (ROS) production, cellular senescence (e.g., the senescence-associated secretory phenotype, SASP), and chronic inflammation. These processes drive metabolic inflexibility across multiple organs and tissues (adipose tissue, liver, heart, and skeletal muscle), ultimately contributing to organ damage and metabolic diseases (e.g., obesity, ageing, and associated disorders). By contrast, targeted nutrient-based interventions, including essential amino acids (EAAs), curcumin, polyphenols, and other bioactive food-derived compounds, can improve mitochondrial function, lower ROS levels, limit senescence, and attenuate chronic inflammation. Collectively, these effects support metabolic flexibility at the tissue/organ level and improve whole-body metabolic health, reinforcing the concept that appropriately designed nutritional strategies can function as pharmacological-like tools to prevent or reverse metabolic dysfunction.

Modifying lifestyle and dietary habits is widely recognized as a powerful strategy for counteracting diseases associated with metabolic dysfunction (e.g., obesity and ageing) and represents the first line of intervention (Baskin and Karp, 2025). In the classical biochemical view, food-derived molecules are regarded primarily as substrates for energy production. However, accumulating evidence indicates that specific nutrients actively modulate metabolic health, influencing both disease development and prevention (Ruocco et al., 2021). These bioactive components (i.e., amino acids, short-chain fatty acids, and polyphenols) regulate signaling pathways, interact directly with proteins, and shape the gut microbiota (Ryan and Seeley, 2013; Menichetti et al., 2025). Consistently, more than 139,000 dietary compounds have been identified with potential biological activity, and approximately 2,000 are already employed for pharmacological purposes (Menichetti et al., 2025). In other words, owing to their mechanisms of action, nutrients may be closer to drugs than to the simple energy substrates traditionally defined in nutritional biochemistry. Together, these insights emphasize that the qualitative composition of the diet, beyond caloric content, plays a crucial role in determining whole-body metabolism, longevity, and health span (Figure 1).

Ageing and obesity are associated with several chronic diseases, including cardiovascular disorders, type 2 diabetes (T2D), leaky gut, metabolic dysfunction-associated steatotic liver disease (MASLD), and cancer. Both conditions share hallmark features such as systemic inflammation, reduced mitochondrial activity, excess reactive oxygen species (ROS) production, and impaired metabolic flexibility across organs and tissues (Bratic and Larsson, 2013; Muoio, 2014). These common features suggest that both conditions arise from overlapping cellular and molecular pathways regulating metabolic health. Moreover, long-term obesity has been associated with the expression of molecular ageing signatures during young adulthood in both females and males, reinforcing the view that obesity may constitute a state of accelerated biological ageing (Correa-Burrows et al., 2025). Metabolic homeostasis depends on a highly integrated network of signaling pathways, and perturbations in any component of this system can propagate across tissues, ultimately leading to multi-organ dysfunction (Sung et al., 2023). In this context, the chronic consumption of a Western diet rich in processed foods, sugars, and unhealthy fats promotes chronic inflammation in multiple organs (Clemente-Suárez et al., 2023), driving adipose tissue expansion and dysfunction, factors that *The Lancet Commission* identified as key determinants of the clinical manifestations of obesity (Rubino et al., 2025). In turn, the resulting metabolic disturbances impair nutrient utilization (i.e., fatty acids, amino acids, and glucose) and contribute to the development of obesity-related diseases (Sung et al., 2023) (Figure 1).

### Beyond calories: nutrients as metabolic modulators

Challenging the notion that “a calorie is just a calorie,” this Research Topic examines how nutrient quality shapes metabolic health and contributes to the prevention or mitigation of metabolic diseases. Despite increasing recognition of the therapeutic potential of nutrients, mechanistic understanding remains incomplete and is currently the subject of extensive research. The bibliometric analysis included in this Research Topic confirms the rapid expansion of studies in this area. Notably, citation burst analysis in the field of vitamin D reveals a shift from an early emphasis on correlation-based studies (e.g., associations between vitamin D levels and obesity or the effects of dietary calcium supplementation) toward investigations exploring causal relationships between obesity and vitamin D deficiency, as well as the preventive or therapeutic effects of vitamin D supplementation on metabolic diseases (Song et al., 2024).

Substantial inter- and intra-individual variability in nutrient metabolism, shaped by sex, age, genetics, and physiological state, is critical for understanding metabolic responses to nutritional interventions. Such variability provides key insights into the molecular mechanisms through which dietary restriction (DR) may influence lifespan (Mihaylova et al., 2023). In this context, Ruocco et al. discuss DR, long considered a universal “magic bullet” for longevity. Human studies, including the CALERIE trial, show that moderate DR improves cardiometabolic health and modulates pathways related to proteostasis, DNA repair, mitochondrial function, and inflammation while revealing links between cellular senescence and metabolic health (Kitzman et al., 2016; Aversa et al., 2024). However, DR responses vary markedly, genetic background can outweigh the intervention itself, and sex-specific factors critically shape lifespan outcomes (Di Francesco et al., 2024; Ruocco et al., 2024). Together, these findings underscore the necessity of personalized nutritional interventions that account for biological variability to effectively promote health span (Figure 1).

### Nutritional interventions as therapeutic tools against obesity and ageing

Building on previous evidence demonstrating the preventive and therapeutic effects of essential amino acids (EAAs) on obesity and insulin resistance (Ruocco et al., 2020), Spataro et al. (2025) show that EAAs can also counteract obesity-induced chronic gut inflammation. Whereas mice fed a high-fat diet (HFD) develop obesity and T2D, associated with impaired intestinal barrier integrity, replacing dietary protein with EAAs reduces weight gain, ameliorates glucose homeostasis, and protects against gut dysfunction. These beneficial effects are mediated by enhanced mitochondrial activity, stimulation of antioxidant defenses, and reduction of gut inflammation, in line with findings showing that dietary EAAs modulate metabolism by acting directly on peripheral tissues such as muscle, adipose tissue, heart, and liver (D’Antona et al., 2016; Tedesco et al., 2018; Ragni et al., 2023).

Consistent with the central role of mitochondria in metabolic homeostasis, Segala et al. (2025) show that EAA supplementation (named α5 mixture) stimulates mitochondrial function and ameliorates MASLD. In mice fed a Western-type high-fat, high-sugar diet, α5 supplementation reduces hepatic steatosis and fibrosis and alleviates MASLD-associated anxiety-like behaviors (Segala et al., 2025).

Ding et al. (2025) also provide a comprehensive overview of nutrients and natural products as anti-ageing therapeutics. Several compounds, including resveratrol, curcumin, apigenin, epigallocatechin-3-gallate (EGCG), coenzyme Q10, and flavonoids such as quercetin and kaempferol, enhance mitochondrial function, reduce ROS production, and inhibit pro-ageing pathways. In line with this, Matin et al. (2025) show that ginger supplementation, whose antioxidant activity derives largely from its phenolic compounds, counteracts age-related oxidative stress in the mouse liver, restoring antioxidant capacity in a dose- and ageing-dependent manner. This aligns with the previous work demonstrating that replacing saturated fats with extra-virgin olive oil enriched in polyphenols improves cardiometabolic and hepatic health (Ruocco et al., 2022).

Ageing, as well as obesity, is accompanied by metabolic alterations that drive cellular senescence. Consequently, targeting senescence has emerged as a promising strategy to counteract these processes and improve metabolic health (Childs et al., 2015; Zumerle et al., 2024). Accordingly, He et al. (2025) report that ageing induces senescence in tongue mucosa and causes disruptions to amino acid and carbohydrate metabolism. Carnosine levels decline with age in both mice and humans, suggesting its potential as a biomarker of ageing, whereas carnosine supplementation attenuates oxidative stress-induced senescence and lowers p21 and β-galactosidase expression.

Accumulating evidence indicates that cellular senescence is a significant risk factor for atherosclerosis, a condition closely linked to both ageing and obesity. Tao et al. (2025) show that sodium nitrate supplementation reverses atherosclerotic lesions in apolipoprotein E-knockout mice fed HFD, a classical preclinical model of atherosclerosis, while reducing vascular inflammation and preventing senescence. These effects are mediated through modulation of the miR-34a–FGF21 axis, highlighting how nutrient-derived interventions can exert anti-senescent and vasculo-protective actions.

Together, these findings reinforce the concept that specific nutrient compositions can act as metabolic modulators by restoring mitochondrial function, enhancing antioxidant defenses, reducing senescence, and lowering inflammation across organs and tissues, thereby preventing or reversing metabolic disturbances associated with obesity and ageing (Figure 1).

### Nutritional interventions as therapeutic tools against cancer

Obesity and ageing are also closely associated with cancer development, a condition characterized by mitochondrial dysfunction and metabolic reprogramming that drive tumor cells toward aerobic glycolysis (i.e., Warburg effect) and amino acid addiction (Ragni et al., 2022a). Branched chain amino acids (BCAAs) can fuel tumor growth, raising concerns about their supplementation in nutrient-based anti-cancer strategies. In this Research Topic, Zhou et al. (2025) highlight that many tumors undergo BCAA metabolic rewiring, with some cancers overexpressing key catabolic enzymes and others exhibiting elevated BCAA levels that promote mechanistic target of rapamycin activation. Notably, BCAA supplementation improves survival in hepatocellular carcinoma, whereas colorectal cancers display inhibition of BCAA catabolism. However, Ragni et al. demonstrated that an EAA-enriched diet impairs tumor growth in mice by stimulating BCAA catabolism, restoring metabolic flexibility, and promoting apoptosis selectively in cancer cells (Ragni et al., 2022b). These findings underscore the context-dependent nature of amino acid metabolism, which varies according to tumor type and reflects the inherent complexity of amino acid metabolic pathways.

Accordingly, Akinlalu et al. (2025) comprehensively review the cancer-specific and often exclusive metabolic roles of individual amino acids, proposing tailored supplementation or withdrawal strategies, as well as targeting amino acid transporters. They further argue that mapping amino acid metabolic heterogeneity across cancer types may yield diagnostic biomarkers and guide precision nutrition-based therapies. This is confirmed by Liu et al. (2024), who report in a nested case–control study that higher serum taurine correlates with reduced overall cancer risk, elevated S-adenosylmethionine (SAM) with increased gastrointestinal cancer incidence, and cysteine with cancer risk in women. However, Bayesian kernel machine regression revealed that the combined mixture of taurine, SAM, and cysteine was negatively associated with cancer risk, indicating a complex, non-linear interaction among these metabolites.

Nutritional strategies may also support the management of cancer-associated conditions such as cachexia, a disorder characterized by muscle wasting, inflammation, and metabolic dysfunction. Faiad et al. (2025) highlight that early, individualized nutritional interventions, particularly high protein intake and anabolic amino acids (e.g., BCAAs), can help attenuate muscle loss and inflammation, especially when combined with supplements such as arginine, omega-3 fatty acids, glutamine, and β-hydroxy-β-methylbutyrate. However, clinical evidence supporting the use of carnitine, omega-3 fatty acids, or antioxidants such as resveratrol, curcumin, and EGCG when administered individually remains limited and inconclusive.

## Conclusion

Building on the concepts summarized in this Research Topic, it is increasingly clear that treating obesity and ageing as whole conditions, rather than as clusters of individual complications, may offer a more effective strategy for improving health span (Garmany et al., 2021; Rubino et al., 2025). Within this framework, nutrient-based interventions, by targeting mitochondrial function, antioxidant and anti-inflammatory pathways, and cellular senescence, represent powerful therapeutic tools, either as alternatives or complements to pharmacological treatments, which, despite their efficacy, are often limited by adverse side effects. This is particularly relevant given the rapid rise of incretin-based drugs, such as GLP-1R agonist (i.e., semaglutide), GLP-1R/GIPR dual agonists (i.e., tirzepatide), or next-generation GLP-1R/GIPR/GlucagonR triple agonists (i.e., retatrutide), which exhibit unprecedented clinical efficacy in metabolic disease management. Notably, evidence that tirzepatide induces a thermogenic-like amino acid signature in brown adipose tissue (i.e., increase in energy expenditure), similar to that observed with nutrient-based interventions such as EAA feeding, highlights a mechanistic convergence between drugs and nutrient modulation of metabolism (Samms et al., 2022; Ruocco et al., 2023). Yet, incretin-based therapies pose nutritional challenges, including lean mass loss, underscoring the need for tailored dietary strategies encompassing adequate protein intake, amino acid support, and resistance training (Ben-Porat et al., 2025). Altogether, these insights emphasize the importance of integrating personalized nutritional approaches with pharmacological treatments to optimize metabolic health and promote longevity.
